# Posterior Sagittal Anorectoplasty for Acquired Imperforate Anus Complicated by Anorectal Necrosis: A Case Report

**DOI:** 10.3390/children9040454

**Published:** 2022-03-23

**Authors:** Yea-Ling Chen, Yu-Tsun Su, Ming-Lun Yeh, Yung-Ning Yang, Ching-Chung Tsai, Po-Jui Ko

**Affiliations:** 1 Department of Pediatrics, E-Da Dachang Hospital, No. 305, Dachang 1st Road, Sanmin District, Kaohsiung City 80794, Taiwan; amystella2001@yahoo.com.tw; 2 Department of Pediatrics, E-Da Hospital, No. 1, Yi-Da Road, Yan-Chao District, Kaohsiung City 82445, Taiwan; suyutsun@yahoo.com.tw (Y.-T.S.); ed106132@edah.org.tw (Y.-N.Y.); 3 School of Medicine, I-Shou University, No. 8, Yi-Da Road, Yan-Chao District, Kaohsiung City 82445, Taiwan; 4 Department of Surgery, Division of Pediatric Surgery, E-Da Hospital, No. 1, Yi-Da Road, Yan-Chao District, Kaohsiung City 82445, Taiwan; ed103434@edah.org.tw

**Keywords:** posterior sagittal anorectoplasty, acquired imperforate anus, anorectal necrosis

## Abstract

Anorectal necrosis is an uncommon lethal disease in children, characterized by necrosis of the mucosa of the anus and rectum. The difference between anorectal necrosis and Fournier’s gangrene is that anorectal necrosis does not affect the genital organs. The treatment for anorectal necrosis includes debridement of the anus, colostomy, and the use of broad-spectrum antibiotics. However, anorectal necrosis may lead to anal stricture, anal malfunction, or even acquired atresia of the anus. There is no consensus on the treatment for acquired imperforate anus. Herein, we report a case of a four-month-old boy with acquired imperforate anus complicated by anorectal necrosis. We describe our experience performing posterior sagittal anorectoplasty to reconstruct a neo-anus in such a rare case.

## 1. Introduction

Both anorectal necrosis and Fournier’s gangrene are necrotizing fasciitis that leads to the formation of gangrenous tissue in the perineum and perianal area. The main difference between these two diseases lies in their distribution. As against Fournier’s gangrene, anorectal necrosis does not involve genital organs and is mainly characterized by necrosis of the anal and rectal mucosa [1,2,3].

Anorectal necrosis affecting children is rarely reported in the literature. Fournier’s gangrene is an uncommon disease that can affect patients from all age groups, predominantly adults aged between 20 and 50 years. It is also rarely found in children. Both anorectal necrosis and Fournier’s gangrene have high associated mortality and morbidity rates [1,3,4].

As anorectal necrosis involves anus and rectal mucosa, it may cause anal stenosis, loss of anal function, or even cause acquired anal atresia [1]. Herein we reported a case of anal necrosis complicated by anorectal necrosis. His anus cannot be preserved in wound healing, which eventually causes acquired imperforate anus. Acquired imperforate anus occurs rarely, and currently, there is no consensus regarding the treatment of acquired imperforate anus. Hence, we present our experience of performing posterior sagittal anorectoplasty to create a neo-anus to treat a child with an acquired imperforate anus.

## 2. Case Presentation

**Figure 1:**
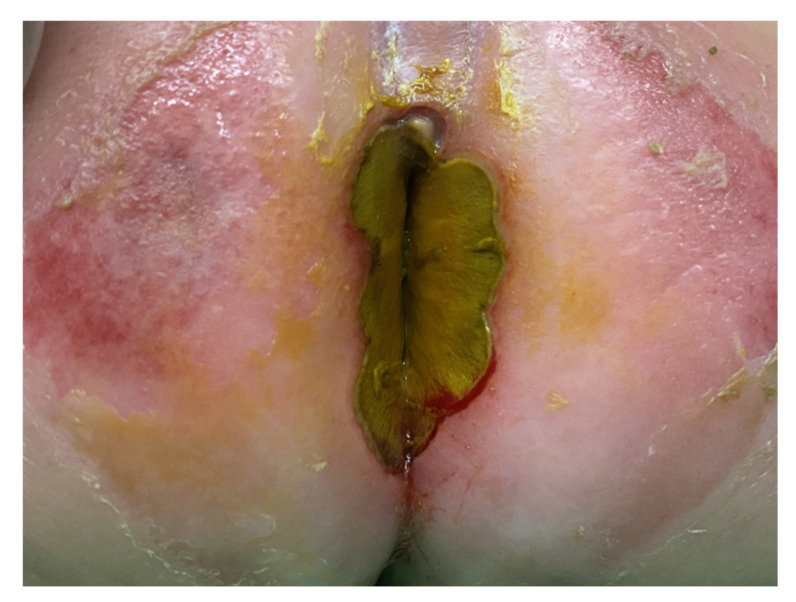
There was a gangrenous change on the skin near the anus.

A four-month-old boy had a fever of up to 38 °C for about six days. Diarrhea episodes were noted 7~8 times per day once the patient started developing a fever. He was admitted due to poor activity under the impression of infectious enterocolitis and dehydration. He had a history of hypospadias but had not received an invasive intervention. He did not have a history of recent trauma, insect bite, steroids use, hospitalization, or immunodeficiency. His vital signs at admission were as follows: body temperature: 36.9 °C, blood pressure: 101/56 mmHg, heart rate: 146 bpm; respiratory rate: 36 respirations/min. Laboratory tests revealed a white blood count of 1980/μL, hemoglobin of 9.8 g/dL, lymphocyte of 50%, band neutrophil of 10%, monocyte of 10%, myelocyte of 3%, metamyelocyte of 7%, typical lymphocyte of 5%, neutrophil of 10%, eosinophil count of 5%, and platelet count of 27,000/μL. Moreover, C-reactive protein level was 143.87 mg/L (normal: <5 mg/L), sodium level was 120 mEq/L (normal: 136–145 mEq/L), and potassium of 3.4 mEq/L (normal: 3.5–5.1 mEq/L) were also noted. Physical examination showed a gangrenous change on the skin near the anus. However, the lower abdomen or genital organs were not involved (Figure 1).

Teicoplanin, Meropenem, and Clindamycin were prescribed. However, despite aggressive treatment and resuscitation, consciousness change, poor activity, and progressive perineal necrotic change were still noted two days later. Hence, emergency surgery was performed for debridement and sigmoid colostomy. Pathology of perineal tissue revealed extensive necrosis, fibrinous deposition, and bacterial colonies. Wound culture revealed *Pseudomonas aeruginosa* infection. Antibiotics were changed to ceftazidime and gentamicin. Subsequently, his leukopenia and high C-reactive protein improved gradually. The wound was packed with wet gauze three times a day initially and changed to Ag-aquacel daily later. As his condition stabilized, he was discharged 15 days after the operation.

**Figure 2:**
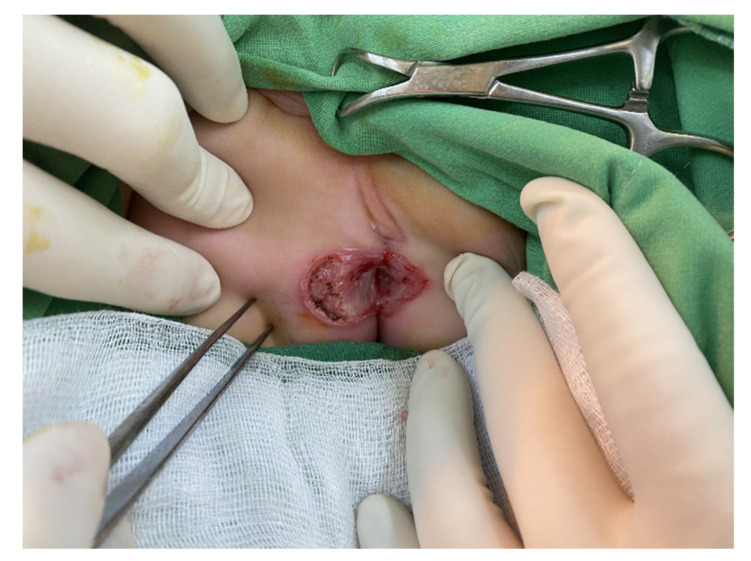
Polypoid granulation tissue was noted on the right buttock, 9 o’clock, with a skin defect.

After discharge, the wound around the anus was continuously packed with Ag-aquacel, and anal dilator #10 was used daily to keep the orifice of the anus patent. Another wound debridement was performed two months later due to a polypoid granulation tissue in the wound (Figure 2).

**Figure 3:**
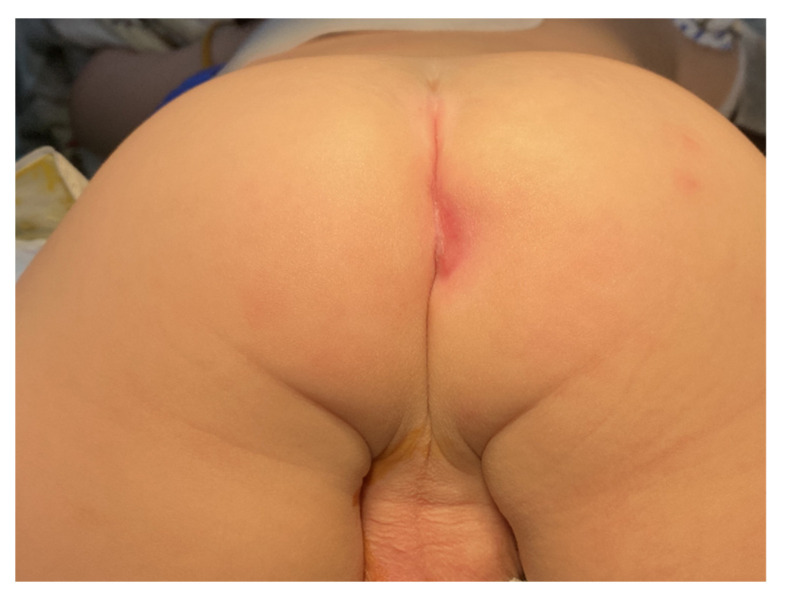
After stopping anal dilator use, the patient’s perianal wound healed well and led to the formation of imperforate anus.

The pathology of that granulation tissue revealed acute suppurative inflammation and necrotic tissue. However, the wound did not heal even after two more months of wound care. Therefore, we let the wound heal by itself, and the anus turned into an acquired imperforate anus spontaneously 5 months after the first debridement (Figure 3).

Subsequently, posterior sagittal anorectoplasty was performed 7 months later to reconstruct the anus for this child. A posterior midline incision was made from the coccyx to the anus, and the distal end of the rectum was found after vertical dissection. Unlike congenital high type imperforate anus, the distance between the rectal pouch and the anus was short. The anatomical structure in that area was relatively normal. We carried out the operation similar to the procedures for the patients with congenital high type imperforate anus. The rectal pouch was sutured and fixed to the skin to create neo-anus. Subsequently, the soft tissue above the anus was repaired layer by layer, according to the anatomical structure before the vertical dissection. Stricture of the neo-anus was noted in the clinics. Another operation was performed 9 months later to release the scar tissue of the suture line. Regular dilatation of the anus using anal dilator #10 everyday by the parents was performed. Meanwhile, we also asked the parents to inject his stool into distal colostomy to check the function of the rectum and anus. Stool passage could be noted from the neo-anus after injecting the stool. His colostomy was closed smoothly one year after the first operation. His postoperative recovery was smooth and uneventful, and his stool passed through neo-anus smoothly thereafter. However, the patient is still less than two years old and the parents have not yet provided the child with toilet training. Therefore, it remains unclear whether the child is continent.

## 3. Discussion

Anorectal necrosis is usually related to leukemia. Wide incision and drainage of perianal abscesses in patients with acute leukemia or uncontrolled chronic leukemia may result in anorectal necrosis [5]. In addition, anorectal necrosis was reported to be caused by a post-traumatic infection in the rectal mucosa following an injury caused by the use of a rectal thermometer or induced by injection sclerotherapy for hemorrhoids [6,7]. About bacterial infection, *Clostridial* organisms, non-*Clostridial* organisms, and *Pseudomonas* were considered key species linked to anorectal necrosis [8,9]. Moreover, *Streptococcus pyogenes* was also reportedly related to rectal necrosis [10]. Fournier’s gangrene may be caused by certain pathogens, including *Escherichia coli, Pseudomonas aeruginosa, Enterococci, Klebsiella pneumonia, Proteus mirabilis, Clostridium* spp., *Bacteroides fragilis, Streptococcus pyogenes*, and *Peptostreptococcus* [2,3,11]. Our patient did not have a history of any underlying disease or trauma history, and his anorectal necrosis was related to *Pseudomonas aeruginosa* infection.

There is a dearth of literature on the treatment of anorectal necrosis and Fournier’s gangrene. The common treatment for both diseases includes surgical debridement, colostomy, and dosing with broad-spectrum antibiotics [1]. In addition, there is little consensus on the surgical management of anorectal necrosis. In this case, the treatment for anorectal necrosis included administration of broad-spectrum antibiotics, a diverting colostomy, and surgical debridement of devitalized tissue.

Kremer et al. inserted a silicon tube into the mucus fistula and brought it out through the anus. The rectal mucosa was sutured and fixed to the tube. The mucosa of their patient became viable later [1]. In our patient, the mucosa of the anus showed necrotic change and subsequently disappeared. We tried preserving the anus by inserting the anal dilator once daily. However, the wound did not heal spontaneously, and the mucosa of the anus could not regrow even four months later. Therefore, we stopped using the dilator, and the wound healed gradually. Subsequently, the anal opening of the anus closed completely. Finally, it turned into an acquired imperforate anus. The posterior sagittal anorectoplasty is a standard procedure for the congenital imperforate anus, and we used this procedure to reconstruct a neo-anus in this child. Kamat and Nuragal described a case of a 35-year-old man who had an acquired imperforate anus complicating a posterior urethral stricture due to trauma in a severe vehicular crash. They used posterior sagittal anorectoplasty to manage acquired imperforate anus [12]. There is little literature regarding the acquired imperforate anus and its management. Hence, we believe that our experience would be valuable for surgeons to deal with similar cases in the future.

## 4. Conclusions

We report a rare case of acquired imperforate anus complicated by anorectal necrosis and describe our experience of performing posterior sagittal anorectoplasty to reconstruct a neo-anus in a child.

## Data Availability

Not applicable.
